# Trust in community as a predictor of public health measure adherence: Insights from a national Canadian survey

**DOI:** 10.14745/ccdr.v51i101112a05

**Published:** 2025-12-12

**Authors:** Neil Seeman, Justin Trent, Kumar Murty

**Affiliations:** 1 Institute of Health Policy, Management and Evaluation, Dalla Lana School of Public Health, University of Toronto, Toronto, ON; 2 Fields Institute for Research in Mathematical Sciences, Toronto, ON; 3 Department of Mathematics, University of Toronto, Toronto, ON

**Keywords:** public health measures, trust in community, adherence to guidelines, predictive modelling, community resilience

## Abstract

**Background:**

Public health models often lack comprehensive behavioural data, leading to inaccurate predictions about the spread of disease and insufficient information about how to effectively build and sustain adherence to changing public health protocols.

**Objective:**

The current study addresses this lack of comprehensive behavioural data by examining the role of trust as a predictor of adherence to public health measures.

**Methods:**

Data were collected from an online Web intercept survey of 3,021 randomly engaged Canadians aged 16 years and older, analyzing factors such as gender, education and sources of COVID-19 information in relation to adherence to public health guidelines.

**Results:**

Trust, respecting someone’s expertise sufficiently to be willing to accept their counsel, emerged as a potent predictor of adherence to public health measures, highlighting the significance of trust in shaping community engagement; further, community-level adherence was found to predict anticipated future adherence.

**Conclusion:**

This study emphasizes the critical role of trust, especially at the community level, in the success of public health measures, and proposes integrating trust measurement into public health models of compliance and resistance.

## Introduction

The COVID-19 pandemic underscored the critical importance of public adherence to health measures in managing public health crises. Understanding the factors that influence this adherence is essential for developing effective strategies to address future health emergencies. This study aims to explore the relationship between trust in the community, healthcare providers and political communications and adherence to public health and social measures (PHSMs) in Canada.

Previous research has highlighted the role of trust in government and health authorities in promoting adherence to public health guidelines (1–4); however, less attention has been directed toward understanding the role of community trust in this context. This study seeks to fill this gap by examining how trust affects both past and future adherence to PHSMs.

The primary objective of this study was to improve the identification of factors such as age, education, region, information sources and community engagement, that affect adherence to health measures, providing public health authorities a clearer picture of the underlying drivers of compliance at both individual and community levels. Additionally, it aimed to gather data to assist in predicting instances of low adherence to PHSMs in future public health crises by considering community-specific influences. Using predictive modelling techniques, the study sought to generate reliable models that can help forecast non-adherence scenarios within different community contexts, allowing public health authorities to design targeted interventions that mitigate risk in critical areas and strengthen community resilience.

## Methods

An online survey was conducted of 3,021 randomly selected participants across Canada, all aged 16 years and older. This survey formed part of a broader international study that included 10 additional countries, resulting in a total sample size of 22,015 respondents.

Regression and classification models were utilized to estimate the impact of trust, location and demographic variables on individual and community adherence to PHSMs in Canada. Statistical analysis was employed to identify outliers within specific sub-groups. To model the relationship between trust and adherence, response survey scales were transformed into numeric values, and trust and adherence scores were calculated for each respondent. Average scores were then computed across demographic groups, such as gender, age, region, city and a combination thereof, provided each group included at least 50 respondents. This was initially analyzed using simple linear regression, which revealed a surprisingly strong relationship between trust and adherence (R^2^>0.84). Predicting individual adherence proved more complex. However, the analysis indicated that individual adherence can be effectively predicted as a binary outcome using a gradient-boosted decision tree classification model using dimensionality reduction of metadata and with further data engineering to refine urban-rural classifications and city size variables. Moreover, this approach appears particularly adept at forecasting shifts in adherence behaviour, such as high past adherence followed by low anticipated future adherence, and vice versa.

The survey instrument was designed to assess the following: 1) trust in community; 2) trust in healthcare providers; 3) trust in political communications; 4) past adherence to PHSMs; 5) anticipated future adherence to PHSMs; and 6) demographic information, including age, gender, education level and primary information sources.

The analysis began with a review of Organisation for Economic Co-operation and Development and Statistics Canada reports on trust, then scanned both grey literature and peer-reviewed publications from the previous three years that mentioned “public trust” in the abstracts. This process helped in the identification of key elements of institutional trust (public trust in institutions) and interpersonal trust (public trust in community) (5,6).

Sample questions were developed based on the literature review. These questions were refined using principles of construct validity, reliability, objectivity and credibility (7), and by drawing on relevant experience from previous COVID-19-related surveys deployed in Ontario and the United States with the same sampling methodology (8,9).

These questions were further refined through feedback from a network of researchers and infectious disease modelers from the Mathematics for Public Health Network of the Fields Institute for Research in Mathematical Sciences. This network represents over a dozen Canadian universities and includes international collaborators from institutes in France, the United Kingdom, Brazil, the United States, Japan and China. Although the survey instrument was deployed in 11 countries, the findings presented here are restricted to Canada, as the literature review could not conclusively establish cross-national transferability of the selected trust elements ((10)).

A pilot study, involving 500 completed surveys, was conducted during the week of September 20, 2022. The regional meta-data, as well as self-reported data on age and gender, were cross-validated with Statistics Canada data (11). No changes to the collection process or the question set were made following the pilot. The full data collection proceeded in two waves: from October 5 to 21, 2022 and from November 16 to December 11, 2022.

As expected with a survey of this nature, where anonymous potential respondents encounter the survey while searching for other information, a modest percentage of participants (19.2%) who opted in to the survey completed all questions. Despite this relatively low completion rate as compared to some incentive-based surveys, it is contended that this method offers data quality advantages over other online non-probability sampling techniques involving actively recruited and/or compensated respondents. Under the sampling approach used, no incentives were offered (eliminating incentive bias), and participants were able to exit the survey at any time. Since the survey sites did not have ad tracking pixels, ad block technology did not reduce the size or diversity of the sample. These techniques seek to reduce self-selection bias, recruitment bias, social desirability bias, acquiescence bias and online coverage bias (9). Further details on survey completion rates, regional, gender and age breakdowns are available in **Appendix** and upon request.

## Results

The findings revealed several key insights into the relationship between trust and adherence to PHSMs.

### Outsized impact of trust in community

The survey indicated that 88.4% of respondents who strongly believed that their community members were diligently following PHSMs reported adhering to these measures themselves (89.7% when adjusted for age, gender and province/territory). In stark contrast, only 30.1% of respondents who perceived their community as rarely following PHSMs reported similar adherence (28.5%, when similarly reweighted). This pattern demonstrates a clear, proportional decline in adherence as trust in community compliance decreases. Similar trends were observed in the respondents’ trust in healthcare providers and political communications.

### Low education, low adherence

Across age groups, regions and information sources, those reporting primary school (or below) as their highest level of formal educational attainment were consistently among the least likely to adhere to PHSMs.

### Complex interplay of age, region and gender

Overall, Canadian seniors (aged 65 years and older) were among the most likely to adhere to PHSMs; however, in certain provinces, adherence among seniors was below both the national and provincial averages. Generally, young males were among the least likely to adhere. Yet, in select urban areas, this demographic reported adherence levels well above the municipal average.

### Role of information sources

Respondents who reported podcasts or radio as their primary source for COVID-19 information showed consistently, and often significantly, lower adherence to PHSMs across almost all age groups and regions. The impact on PHSM adherence among those relying on other information sources varied in both direction (positive or negative) and magnitude, depending on age group, education level and region.

### Trust data can facilitate predictive modelling

Results suggest that knowledge of trust in community, healthcare practitioners and political communications was sufficient to make reasonably accurate group-level predictions of adherence levels across a wide range of age, region and gender combinations. Further analysis indicates that individual adherence could be predicted with reasonable accuracy as a binary outcome, using the higher-dimensional data collected along with additional feature engineering.

## Discussion

These findings highlight the complex relationship between trust and adherence to PHSMs, with community trust emerging as the most influential predictor of adherence. This finding has significant implications for public health strategies, suggesting that efforts to build and maintain trust at the community-level are critical for ensuring compliance during health crises.

The variations in trust and adherence based on geography, age, education and information sources emphasize that a one-size-fits-all approach to public health messaging is unlikely to be effective. Instead, strategies that are tailored to local contexts and community dynamics, such as engaging local leaders and partnering with trusted community figures may prove more successful in promoting adherence to PHSMs (aligning with results seen in Barrett *et al.*) (9). The strong correlation between trust and adherence highlights the importance of understanding trust dynamics to pinpoint areas where adherence may be low, allowing public health authorities to target their efforts more effectively.

Furthermore, the nuanced demographic variations observed in the study indicate that simply attributing non-adherence to factors like the negative influence of social media or demographic stereotypes (e.g., the “angry young male”) is counterproductive. Public health authorities must work to understand the complex interplay of age, location, gender and information sources.

The success of the predictive model deployed across a range of demographics and communities highlights the potential value of measuring trust as a tool for anticipating PHSMs adherence. This could enable public health authorities to identify areas or groups where compliance is likely to be low and implement targeted interventions designed to address specific trust deficits, ultimately improving public health outcomes during future crises.

## Limitations

This study has several limitations that should be considered when interpreting the results: 1) self-reported data: a reliance on self-reported adherence may be subject to social desirability bias; 2) cross-sectional design: this study provides a snapshot of trust and adherence but cannot establish causal relationships or track changes over time; 3) generalizability: while this survey was deployed in multiple countries, the findings here may not be universally applicable to all public health crises or cultural contexts; and 4) this survey did not include questions related to race/ethnicity or income-level.

While other studies have suggested that race/ethnicity or income-level play a role in adherence, these were not included as the study’s goal was to use a single question set across all 11 countries covered by this survey (translated to the local languages where applicable) and the income brackets as well as ethnic make-up shifted considerably from country to country.

### Future research

Future research could address these limitations through 1) longitudinal studies to track changes in trust and adherence over time, 2) experimental designs to explore causal relationships between trust and adherence, 3) in-depth qualitative studies to assess the nuances of community trust in different contexts; 4) studies focusing on specific demographic groups or regions to develop more targeted interventions; and 5) comparing and contrasting results in Canada with those of other countries surveyed, sensitive to the challenges in the cross-national transferability of the survey questions.

## Conclusion

Trust was found to be closely tied to adherence, with community trust emerging as a particularly strong predictor. This pattern persisted across multiple demographic groups and regions, suggesting that local trust plays an even more critical role in compliance than trust in healthcare providers or political figures.

This study improved the identification of factors influencing adherence by highlighting the significance of demographic variables such as education, age, gender and region, as well as preferred information sources. Lower education levels and reliance on certain media (e.g., podcasts and radio) were associated with reduced adherence. These findings point to the need for targeted public health messaging that addresses the specific concerns and trust deficits within different population subgroups.

Finally, the data collected proved effective for predicting instances of low adherence. The models used, which incorporated trust data along with demographic variables, enabled accurate group-level predictions, offering a potential tool for public health authorities to anticipate and address areas of low compliance in future crises.

In conclusion, trust, particularly at the community-level, is a critical lever for ensuring public health compliance. To maximize adherence in future health emergencies, public health strategies should prioritize building and maintaining trust within local communities, measuring trust at the community-level and tailoring communications to address the demographic and regional variations in both trust and behaviour. To serve these goals, trust measurement can be embedded in public health models of compliance and resistance. By doing so, authorities can better predict and mitigate areas of low adherence, enhancing the overall effectiveness of public health interventions.

## Appendix

**Table A1:** Completion rates and demographic characteristics of survey respondents^a,b,c^

Characteristic	Completion rate	Population	Surveys
Total study group	19.2%	100%	3,021
**Gender (at age 16 years)**			
Female	19.9%	50.9%	30.6%
Male	20.0%	48.9%	59.0%
Prefer not to answer	14.6%	0.2%	10.4%
**Age group (years) (for participants aged 16 years and older)**			
16–24	19.4%	14.2%	25.5%
25–34	16.6%	17.4%	17.8%
35–44	17.6%	16.2%	16.3%
45–54	18.2%	14.4%	12.3%
55–64	21.7%	15.4%	12.0%
65 and older	24.5%	22.3%	16.1%
**Region**			
Alberta	18.1%	11.5%	10.3%
British Columbia	16.7%	13.5%	12.1%
Manitoba	22.2%	3.6%	3.6%
New Brunswick	21.9%	2.1%	1.9%
NFL and Labrador	26.8%	1.4%	1.4%
Nova Scotia	20.8%	2.6%	2.8%
Ontario	19.5%	38.5%	42.8%
PEI	15.5%	0.4%	0.4%
Québec	19.7%	23.0%	22.2%
Saskatchewan	19.7%	3.1%	2.4%
Territories	21.7%	0.3%	0.2%

Abbreviations: NFL, Newfoundland and Labrador; PEI, Prince Edward Island

^a^ Both unweighted and reweighted (by age group, gender and province/territory as outlined below) data have been included in the aggregate figures

^b^ Completion rate was significantly lower among respondents who preferred not to provide their gender and notably higher among those whose self-reported age was over 65. As other demographic information was collected at the end of the survey, we cannot comment on the impact of those characteristics on completion

^c^ The proportion of surveys completed by self-reported females and respondents aged 65 and older was significantly lower than population estimates provided by Statistics Canada. Respondents aged 16 to 25 constituted a notably higher proportion of completed surveys compared to their population representation. Representation among other groups aligned with population estimates

### Appendix 2: Questionnaire

This survey is being carried out by the Fields Institute for Research in Mathematical Sciences for the purposes of research. You will not be asked for any personal identifying information. There is no risk to you in filling out this survey and you will not benefit from taking part. If you would like more information about this study you can contact the Fields Institute. This survey has been reviewed by the Human Research Ethics Program at the University of Toronto. By continuing with this survey, you agree to participate in this research study and acknowledge that you are over the age of 15.

**Table:**

Questions	Possible answers
Age	16 to 75+
Are you	Male Female Prefer Not to Answer
Please Select Your Region	[Provinces/Territories in Canada]
**In this survey, we would like to know how you feel about public health policies and the guidelines used to deal with COVID-19.**	
Would most people in your local community follow public health guidelines during a public health crisis?	Yes, almost everyone would follow the guidelines Yes, many would follow the guidelines Maybe half of the population would follow the guidelines No, many would not follow the guidelines No, almost no one would follow the guidelines
Do you feel that **doctors and other health professionals** have communicated useful and reliable information to meet your needs during the COVID public health crisis?	Yes, the information has been completely useful and reliable Yes, the information has been quite useful and reliable The information has been somewhat useful and reliable No, the information has not been useful and reliable No, the information has been wrong and misleading
Do you feel that **political leaders** have communicated useful and reliable information during the COVID-19 public health crisis?	Yes, the information has been completely useful and reliable Yes, the information has been quite useful and reliable The information has been somewhat useful and reliable No, the information has not been useful and reliable No, the information has been wrong and misleading
How much did you follow the health guidelines given **over the past two years** to prevent the spread of the pandemic?	I was very careful to follow all the guidelines I followed most of the guidelines I followed some of the guidelines I did not follow the guidelines
If there is a new health emergency **in the future**, how much would you follow any new health guidelines?	I would follow all the guidelines I would follow most of the guidelines I would follow some of the guidelines I would not follow the guidelines
In the event of a **future disease outbreak**, whose information would you find most useful and believable?	World Health Organization Red Cross/Red Crescent Doctors and Nurses Other Public Health Officials National Government/Health Minister Local Government Military/Police I don’t believe any of the above would provide use useful and believable information
Where do you live?	Rural area/village/small town Medium town/small city Medium city Large city
Which of the below is your **primary source** of information about COVID-19?	International media National media Government Briefings World Health Organization Directly from Friends and Family Social Media (Facebook, Twitter, Instagram, Tik Tok, etc.) Social messengers (e.g. WhatsApp groups, Telegraph, etc.) Radio Podcasts and Blogs TV News
How much schooling have you completed?	No formal education Primary school Secondary school/High school Post-secondary/Vocational training Bachelor's Degree Master's Degree or Higher
